# Genome informatics and vaccine targets in *Corynebacterium urealyticum *using two whole genomes, comparative genomics, and reverse vaccinology

**DOI:** 10.1186/1471-2164-16-S5-S7

**Published:** 2015-05-26

**Authors:** Luis Carlos Guimarães, Siomar de Castro Soares, Eva Trost, Jochen Blom, Rommel Thiago Jucá Ramos, Artur Silva, Debmalya Barh, Vasco Azevedo

**Affiliations:** 1 Department of General Biology, Institute of Biological Sciences, Federal University of Minas Gerais, Avenue Antônio Carlos, 6627, Belo Horizonte, Minas Gerais, Brazil; 2 Department of Preventive Veterinary Medicine, School of Veterinary Medicine, Federal University of Minas Gerais, Avenue Antônio Carlos, 6627, Belo Horizonte, Minas Gerais, Brazil; 3 Institut für Hygiene, Universitätsklinikum Münster, Albert-Schweitzer-Campus 1, Münster, Germany; 4 Bioinformatics and Systems Biology, Justus-Liebig-University Giessen, Ludwigstasse, 23, Giessen, Germany; 5 Department of Genetics, Institute of Biological Sciences, Federal University of Pará, Avenue Augusto Corrêa, 01, Belém, Pará, Brazil; 6 Centre for Genomics and Applied Gene Technology, Institute of Integrative Omics and Applied Biotechnology (IIOAB), Nonakuri, Purba Medinipur, WB-721172, India

**Keywords:** *Corynebacterium urealyticum*, comparative genomics, metabolic pathway, genome plasticity, antigenic targets

## Abstract

**Background:**

*Corynebacterium urealyticum *is an opportunistic pathogen that normally lives on skin and mucous membranes in humans. This high Gram-positive bacteria can cause acute or encrusted cystitis, encrusted pyelitis, and pyelonephritis in immunocompromised patients. The bacteria is multi-drug resistant, and knowledge about the genes that contribute to its virulence is very limited. Two complete genome sequences were used in this comparative genomic study: *C. urealyticum *DSM 7109 and *C. urealyticum *DSM 7111.

**Results:**

We used comparative genomics strategies to compare the two strains, DSM 7109 and DSM 7111, and to analyze their metabolic pathways, genome plasticity, and to predict putative antigenic targets. The genomes of these two strains together encode 2,115 non-redundant coding sequences, 1,823 of which are common to both genomes. We identified 188 strain-specific genes in DSM 7109 and 104 strain-specific genes in DSM 7111. The high number of strain-specific genes may be a result of horizontal gene transfer triggered by the large number of transposons in the genomes of these two strains. Screening for virulence factors revealed the presence of the *spaDEF *operon that encodes pili forming proteins. Therefore, *spaDEF *may play a pivotal role in facilitating the adhesion of the pathogen to the host tissue. Application of the reverse vaccinology method revealed 19 putative antigenic proteins that may be used in future studies as candidate drug or vaccine targets.

**Conclusions:**

The genome features and the presence of virulence factors in genomic islands in the two strains of *C. urealyticum *provide insights in the lifestyle of this opportunistic pathogen and may be useful in developing future therapeutic strategies.

## Background

The species *Corynebacterium urealyticum *was proposed in 1986, but this bacteria was first isolated between June 1983 and March 1984, when four patients were diagnosed with alkaline-encrusted cystitis. The published case report describes the isolates as belonging to the Corynebacterium group D2 [1,2]. Chemotaxonomic studies and 16S rRNA sequence comparisons showed that *C. urealyticum *was more closely related to Corynebacterium lipophilic species such as *Corynebacterium jeikeium*, but could be differentiated from *C. jeikeium *based on its ability to hydrolyze urea [3]. *C. urealyticum *is a Gram-positive, non-spore-forming, aerobic, and slow-growing bacteria. Its cell wall is composed of peptidoglycan, menaquinone, mycolic acids, and cellular fatty acids, which is the common composition of the cell walls of Corynebacterium species [4].

*C. urealyticum *is an opportunistic pathogen commonly isolated from the skin and mucous membranes of hospitalized patients. The pathogen mainly causes acute or encrusted cystitis, encrusted pyelitis, and pyelonephritis [4]. Its urease activity is the main factor that contributes to the ability of *C. urealyticum *to colonize the urinary tract where its presence is associated with alkaline pH and the formation of ammonium magnesium phosphate stones [1,2]. *C. urealyticum *is a multi-drug resistant bacterium and its treatment requires the administration of multiple drugs and additional invasive interventions [4,5]. Currently, the complete genome sequences of only two *C. urealyticum *strains, DSM 7109 and DSM 7111, are publicly available. In these two genomes, the antibiotic resistance genes were located in mobile DNA, suggesting that the multidrug resistance was acquired through horizontal gene transfer [6,7].

In this work, we compared the genome sequences of the two *C. urealyticum *multidrug resistance strains DSM 7109 and DSM 7111, focusing on differences in the gene content and metabolic pathways between the two strains. We also attempted to identify new candidate targets that can be used in the development of drugs or vaccines against this pathogen.

## Methods

### Genome sequences of *C. urealyticum *strains DSM 7109 and DSM 7111

*C. urealyticum *DSM 7109 was isolated from a patient with alkaline-encrusted cystitis and *C. urealyticum *DSM 7111 was isolated from the urine samples of a 9-year-old patient with an ectopic kidney. The genome sequences of both these strains were retrieved from the NCBI GenBank database (http://www.ncbi.nlm.nih.gov/genbank/) [8] [GenBank:NC_010545.1, GenBank: NC_020230.1].

### Bioinformatics analysis

The origin of chromosomal DNA replication (*oriC*) gene was predicted using the Ori-Finder web program [9]. The Ori-Finder prediction was based on a combined process: (i) gene identification involving analysis of base composition through Z-curve method; and, (ii) occurrence of genes frequently close to *oriC*s (distribution of *dnaA *boxes along the genome) [9]. The CRISPRs were predicted using the CRISPRfinder web program [10]. These regions are important because they confer protection against bacteriophages. The comparative analysis was done using the EDGAR web-program that compares genome content based on the calculation of the BLAST score ratio by automatically adjusted cutoff for each selected dataset [11].

### *In silico *identification metabolic pathway construction

The metabolic pathways reconstruction of *C. urealyticum *was performed using the genome sequence file in FASTA format and the genome annotation file in GBK format. Metabolic pathways databases for strains DSM 7109 and DSM 7111 were created using the Pathway Tools 13 software (available at http://bioinformatics.ai.sri.com/ptools/), developed by SRI International [12]. The Pathway Tools software contains algorithms that can predict the metabolic pathways of an organism from its genome by comparing it to a reference pathways database known as the MetaCyc Database [13]. Construction of a metabolic pathways database was done using the BioCyc collection [14].

### Prediction of genome plasticity of *C. urealyticum *DSM 7109 and DSM 7111 strains

Prediction of genomic islands in *C. urealyticum *genomes was done using PIPS software [15]. PIPS detects genome signatures like C+G content, codon usage deviation, high concentrations of virulence factors, hypothetical proteins, the presence of transposases and tRNA flanking sequences, and absence of query regions in non-pathogenic organisms. *C. glutamicum *ATCC 13032 was used as the closely related non-pathogenic species to *C. urealyticum *in PIPS. The BRIG software [16] was used for plasticity comparisons among *C. urealyticum *(DSM 7109 and DSM 7111 strains), *C. pseudotuberculosis *1002, *C. diphtheriae *NCTC 13129, *C. ulcerans *809, and *C. glutamicum *ATCC 13032.

### Prediction of putative antigenic targets of *C. urealyticum*

To identify antigenic targets, we used the strategy described by Barh et al. [17] with modifications. We also adopted the four rules as per the reverse vaccinology strategy of Rappuoli et al. [18] for final selection of the putative vaccine targets. Rule I: consider the antigenic proteins that are either secreted proteins, surface-exposed proteins, or membrane proteins so that they can be exposed to the host, and therefore can be promptly recognized by the host immune system [18]; Rule II: major histocompatibility complex (MHC) I and II binding properties with adhesion probability greater than 0.51 and absence of similarity to host proteins [19]; Rule III: protein conservation among different genomes [19]; and Rule IV: virulence factors are normally encoded within genomic islands [18]. Rule IV does not exclude the targets from Rule III.

SurfG+ software [20] was used to predict targets according to the Rule I. This software classifies proteins according to their subcellular location using the presence or absence of signal peptides, retention signals, and transmembrane helices. To apply Rule II, the proteins predicted by surfG+ were analyzed using the Vaxign software [19]. Because the aim of this work was to identify vaccine candidates, the predicted proteomes were screened for proteins that were potentially antigenic in both strains (Rule III). To achieve this, we used the Artemis Comparison Tool [21] with BLAST alignment comparison files and searched for antigenic proteins that show more than 70% similarity in 70% of their extensions in both strains. Base on Rule IV, we screened the detected antigenic proteins for antigenic targets harbored by shared genomic islands in the two strains.

## Results and discussion

### Genomic architecture and features of *C. urealyticum *strains DSM 7109 and DSM7111

Strains DSM 7109 and DSM 7111 were isolated from patients with alkaline-encrusted cystitis [6,7]. The genomic composition of these two strains is very similar; i.e., both sequences have the same G+C content, coding density, ribosomal RNAs clusters, and clustered regularly interspaced short palindromic repeats (CRISPRs). However, the sizes of the two genomes are different: the DSM 7111 genome contains 2,316,065 bp and is 50 Kb smaller than the DSM 7109 genome with 2,369,219 bp. As expected, the number of coding sequences in the DSM 7111 genome (1,927 protein coding regions) is lower than in the DSM 7109 genome (2,011 protein coding regions) because both genomes have the same coding density. These data indicate a strain-specific difference in the gene repertories in both these isolates. Relevant data and general features from both genome sequences are summarized in Table 1.

**Table 1:** General features of the genomes of *C. urealyticum *strains DSM 7109 and DSM 7111.

Feature	DSM 7109	DSM 7111
Genome size (bp)	2,369,219	2,316,065
C+G content (%)	64.2	64.2
Coding sequences	2,084	2,007
Coding density (%)	90.16	89.53
ribosomal RNAs	3 × (16S - 23S - 5S)	3 × (16S - 23S - 5S)
transfer RNAs	51	54

**Figure 1:**
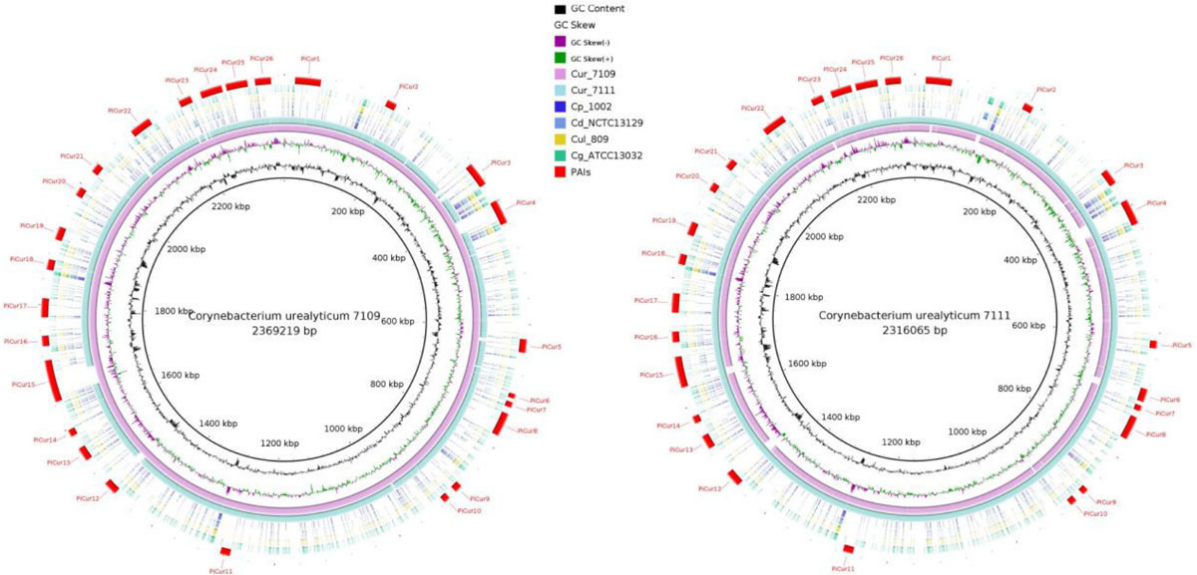
**Comparative genomic maps of *C. urealyticum *strains DSM 7109 and DSM 7111**. The *C. urealyticum *DSM 7109 map (left) used as the reference. The *C. urealyticum *DSM 7111 map (right) used as the reference. From the inner to outer circles on the genome maps: *C. urealyticum *DSM 7109 (Cur_7109) then *C. urealyticum *DSM 7111 (Cur_7111) for the map on the left; *C. urealyticum *DSM 7111 (Cur_7111) then *C. urealyticum *DSM 7109 (Cur_7109) for the map on the right; followed by *C. pseudotuberculosis *1002 (Cp_1002), *C. diphtheriae *NCTC 13129 (Cd_NCTC13129), *C. ulcerans *809 (Cul_809), *C. glutamicum *ATCC 13032 (Cg_ATCC13032). The outermost circles in both maps indicate the 26 genomic islands (PiCur 1-26).

Our analysis of the GC skew [(G-C)/(G+C)] revealed that both genome sequences contained a bi-directional replication mechanism (Figure 1). The origin of the chromosomal replication (*oriC*) gene is located between the replication initiator genes *dnaA *(downstream) and *dnaN *(upstream) and has a size of 843 bp. However, the G/C skew analysis did not confirm that the replication termination site, *dif*, was located at the 180° position from *oriC *[9,22].

CRISPRs are often associated with *cas *genes that normally provide resistance against bacteriophages [23]. One CRISPR region was predicted in each genome (strains DSM 7109 and DSM 7111) using the CRISPRFinder software [10]. Both these regions were flanked by seven *cas *genes. The size of one CRISPR was 28 bp and the consensus sequence was the same for the CRISPRs in both genomes. Each CRISPR was separated by 69 bp (Table 2).

**Table 2:** Structural features of CRISPR loci predicted in *C. urealyticum *strains DSM 7109 and DSM 7111.

Strain	No. of CRISPR loci	No. of cas genes	locus_tag *cas *genes	No. of spacers	CRISPR size	*CRISPR consensus sequence
*Corynebacterium urealyticum *DSM 7109	1	7	cur_1967 cur_1968 cur_1969 cur_1970 cur_1971 cur_1972 cur_1973	69	28 bp	GGCTCATCCCCGCTGGCGCGGGGAGCAC

*Corynebacterium urealyticum *DSM 7111	1	7	CU7111_1887 CU7111_1888 CU7111_1889 CU7111_1890 CU7111_1891 CU7111_1892 CU7111_1893	69	28 bp	GGCTCATCCCCGCTGGCGCGGGGAGCAC

*CRISPR, clustered regularly interspaced short palindromic repeats

### Gene sharing among the two *C. urealyticum *strains

Orthologous genes were detected using the EDGAR software, which defines subsets of genes using the SRV method to predict orthologous genes in prokaryotic genomes [11]. We found that the DSM 7109 and DSM 7111 genomes together encode 2,115 no-redundant coding gene sequences; 1,823 (86.2%) of these coding gene sequences were common to both strains, and 188 and 104 were specific to DSM 7109 and DSM 7111, respectively. Species-specific genes have been linked to niche adaptation of microorganisms. A previous study of 17 *Escherichia coli *strains found that less than 50% of the genes (2,200 genes in a total of 5,000 genes) were shared among these strains [24]. Therefore, we can infer that the DSM 7109 and DSM 7111 genomes are very similar because they share a high proportion of their genes. A previous study of four *Corynebacterium pseudotuberculosis *strains [25] also reported a large number in shared genes; 1,851 (77.9%) genes in a total of 2,377 genes. Clearly, the numbers of core genes are likely to reduce when more strains of *C. urealyticum *are added, as was shown previously by Soares and colleagues in a study of 15 *C. pseudotuberculosis *strains were 1,504 (54.5%) genes in a total of 2,782 genes were shared [26].

### Plasticity of the *C. urealyticum *DSM 7109 and DSM 7111 genomes

Genome plasticity has been used to provide insights into genome evolution through the study of horizontally acquired genomic regions. The transfer of blocks of genes (genomic islands) normally correlates with the acquirement of a given function, like virulence (pathogenicity islands), degradation of secondary compounds (metabolic islands), antibiotic resistance (resistance islands) and symbiotic relationships with Leguminosae (symbiotic islands)[15]. Furthermore, because genomic islands are acquired from a different organism, they are responsible for deviations in genomic signatures such as codon usage and G+C content once they reflect the genomic signature of the donor organism [27]. We used the PIPS software [15] to predict genomic islands in both *C. urealyticum *strains. Twenty-six genomic islands were predicted in each genome (Figure 1). DSM 7109 had 556 genes present in the genomic islands and DSM 7111 had 496 genes. We identified 403 genes in the genomic islands that belonged to the shared genes dataset, meaning that the majority of genes that were acquired by horizontal gene transfer were commonly shared genes.

Previous studies on genomic islands in *C. pseudotuberculosis *and *C. diphtheriae *identified 16 and 52 genomic islands, respectively [26,28]. Only nine of the genes in the genomic islands were shared among *C. urealyticum *strains DSM 7109 and DSM 7111, *C. pseudotuberculosis *1002, and *C. diphtheriae *NCTC 13129 (Table 3). The low number of shared genes among different species in the same genus is expected because their habitats are different and genomic islands are normally acquired through horizontal gene transfer as was shown previously by Perrin and colleagues [29]. Pathogenicity islands contain genes correlated with virulence [27]. Therefore, these genes may be good candidates for the development of vaccines or drugs [15]. When we compared the orthologs of the candidate virulence factors reported in *C. ulcerans *[30] with the genes in the pathogenicity islands in *C. urealyticum *we found only one common gene and this gene was annotated with unknown function (ID: CU7111_1212 for DSM 7111; ID: cur_1230 for DSM 7109).

**Table 3:** Genes present in genomic islands shared by related Corynebacterium species.

*C. urealyticum *DSM 7109	*C. urealyticum *DSM 7111	*C. pseudotuberculosis *1002	*C. diphtheria *NCTC 13129	Gene name	Product
cur_1756	CU7111_1693	Cp1002_1932	DIP2331	-	Putative aldehyde dehydrogenase
cur_1817	CU7111_1751	Cp1002_1909	DIP2133	-	Fe-S oxidoreductase
cur_1897	CU7111_1828	Cp1002_1870	DIP0236	*srtB*	Fimbrial associated sortase (Surface protein transpeptidase)
cur_1898	CU7111_1829	Cp1002_1872	DIP0235	*spaD*	Putative surface-anchored protein (Fimbrial subunit)
cur_1899	CU7111_1830	Cp1002_1874	DIP0233	*srtC*	Fimbrial associated sortase (Surface protein transpeptidase)
cur_1933	CU7111_1856	Cp1002_0132	DIP0247	*tadA*	tRNA-specific adenosine deaminase
cur_1934	CU7111_1857	Cp1002_0131	DIP0246	-	Hypothtical protein
cur_1935	CU7111_1858	Cp1002_0130	DIP0245	*tyrA*	Prephenate dehydrogenase
cur_1939	CU7111_1861	Cp1002_0120	DIP0179	-	Putative dicarboxylate uptake system

The genomes of *C. urealyticum *strains DSM 7109 and DSM 7111, *C. pseudotuberculosis *1002, and *C. diphtheriae *NCTC 13129 were compared.

### Prediction of candidate vaccine targets for *C. urealyticum*

The sub-cellular location of proteins in DSM 7109 and DSM 7111 was predicted using the SurfG+ software [31], which classifies genes into four categories: cytoplasmic, membrane, PSE (putative surface-exposed), and secreted (Table 4). We used the four rules described in the reverse vaccinology strategy (see the Methods section for details) for final selection of putative vaccine targets. According to Rule I, proteins exposed to the host are better candidates because they can be promptly recognized by the immune system; for example, secreted proteins, surface-exposed proteins, and membrane proteins. We predicted 590 and 579 putative candidates for DSM 7109 and DSM 7111, respectively, using Rule I. The encoded proteins were submitted to the Vaxign software [19], which detected 54 and 57 proteins with antigenic properties in DSM 7109 and DSM 7111, respectively. Using Rule III, we considered only proteins that were shared by both strains, which resulted in 46 candidates for both strains. Finally, using Rule IV, we identified 19 proteins that were shared by both strains and that were encoded within genomic islands as vaccine candidates (Table 5). Among these 19 vaccine candidates, six were annotated with a function and a gene name. These proteins have been identified as potential vaccine targets in previous studies [30,32-36], but as yet no tests have been carried out to confirm this.

**Table 4:** Subcellular location of proteins from *C. urealyticum *strains DSM 7109 and DSM 7111.

Feature	DSM 7109	DSM 7111
Cytoplasmic proteins	1431	1356
Membrane proteins	311	302
PSE^a ^proteins	200	198
Secreted proteins	79	79

^a^Putative surface-exposed

**Table 5:** Putative antigenic proteins identified using Vaxign and shared by two *C. urealyticum *strains.

DSM 7109 - Locus_Tag	DSM 7111 - Locus_Tag	Gene name	Subcellular location	Gene product
cur_0025	CU7111_0027	*rpfc*	secreted	Resuscitation-promoting factor RpfC
cur_0151	CU7111_0157	-	secreted	Putative secreted protein
cur_0291	CU7111_0284	-	PSE	Putative surface-anchored protein
cur_0295	CU7111_0288	-	PSE	Hypothetical protein
cur_0527	CU7111_0510	-	PSE	Putative secreted protein
cur_0530	CU7111_0513	*mepA*	secreted	Putative secreted metallopeptidase
cur_1309	CU7111_1290	-	PSE	Hypothetical protein
cur_1319	CU7111_1300	-	PSE	Putative ribonuclease
cur_1350	CU7111_1330	-	secreted	Putative secreted protein
cur_1399	CU7111_1390	*lppS*	PSE	Putative lipoprotein
cur_1604	CU7111_1545	-	secreted	Hypothetical protein
cur_1636	CU7111_1577	-	secreted	Putative secreted protein
cur_1834	CU7111_1766	-	PSE	Hypothetical protein
cur_1842	CU7111_1775	*cmtA*	secreted	Trehalose corynomycolyl transferase A
cur_1896	CU7111_1827	*spaE*	PSE	Putative surface-anchored protein (fimbrial subunit)
cur_1898	CU7111_1829	*spaD*	PSE	Putative surface-anchored protein (fimbrial subunit)
cur_1958	CU7111_1880	-	secreted	Hypothetical protein
cur_1959	CU7111_1881	-	secreted	Hypothetical protein
cur_1980	CU7111_1900	*crcB*	membrane	Putative fluoride ion transporter CrcB

The *rpfC *gene (resuscitation-promoting factor) is a member of a protein family (*rpfA*, *rpfB*, *rpfD*, and *rpfE*) found in Actinobacteria. The protein encoded by *rpfC *plays a role in stimulating resuscitation of dormant cells and in the multiplication of normal viable bacteria. Studies in *Mycobacterium luteus *with a disrupted *rpf *gene were not possible because of the absence of a second functional copy of the gene, suggesting that this protein is essential for normal growth and reduces the lag phase of diluted fast-growers [32,37].

The *mepA *gene (penicillin-insensitive murein endopeptidase) in *E. coli *encodes a protein the cleaves the D-alanyl-meso-2,6-diamino-pimelyl amide bond of peptidoglycans [38]; however, this protein is sensitive to metal-chelating agents such as lipoteichoic acid and deoxyribonucleic acid [39]. Previous studies with metallopeptidases showed that animals infected with *C. pseudotuberculosis *reacted to the mepA protein, while non-infected animals did not. This protein has transmembrane domains, another strong indication that it may make a good molecular vaccine target [33,40].

The *lpps *(lipoprotein) gene encodes a protein that is associated with cell envelopes and has four known lipoprotein functions: (i) structural function (murein lipoproteins); (ii) transport function (substrate-binding proteins of ABC transporters in Gram-positive bacteria); (iii) adhesion function; and (iv) enzymatic function. The lipoprotein present in *C. urealyticum *has an L,D-transpeptidase catalytic domain, which gives the bacteria the ability to resist beta-lactam antibiotics by inhibiting PBPs (penicillin-binding protein) [34,41].

The *cmtA *(trehalose corynomycol transferase) gene encodes a protein that has catalytic function. It plays a role in the transfer of mycolic acids through trehalose monocorynomycolate on the cell wall arabinogalactan to another trehalose monocorynomycolate to produce trehalose dicorynomycolate [42].

The *spaD *and *spaE *genes are part of the *spaDEF *cluster that encodes adhesive pilus structures that are surface-anchored to the cell walls of Corynebacterium where they probably facilitate the adhesion of the pathogen to the host tissue. We detected the *spaF *gene in the DSM 7109 and DSM 7111 genomes, as well as the sortase encoding genes *srtB *and *srtC*. The genome organization of the *spaDEF *cluster in the two *C. urealyticum *strains is similar to cluster organization in *C. diphitheriae *NCTC 13129 and *C. ulcerans *809 and BR-AD22 strains [30,43]. However, the *spaABC *cluster of genes proposed as an essential virulence factor in *C. diphtheria *[35] was absent in both strains of *C. urealyticum *analyzed.

The *crcB *gene encodes a putative membrane protein, important for the reducing the fluoride concentration in cells, thus reducing its toxicity. Fluoride ions reduce cell growth, even when present in millimolar concentrations. Thus, we can infer that *crcB *gene is an efficient resistance mechanism [36].

### Differences in metabolic pathways in the *C. urealyticum *genomes

To predict the metabolic pathways encoded in the DSM 7109 and DSM 7111 genomes, we used the Pathway Tools software (version 13.0) [44], and detected 226 and 271 pathways in DSM 7109 and DSM 7111, respectively. We also identified 942 and 1,116 metabolic reactions for these strains (Table 6).

**Table 6:** Numbers of gene data types in *C. urealyticum *strains DSM 7109 and DSM 7111.

Data type	DSM 7109	DSM 7111
Genes	2082	1998
Pathways	226	271
Metabolic reactions	942	1116
Transport reactions	34	36
Polypeptides	2022	1935
Enzymes	536	543

Comparative analysis of two pathway classes (Biosynthesis and Degradation/Utilization/Assimilation) showed that the DSM 7109 and DSM 7111 genomes had 139 and 174 Biosynthesis pathways, respectively (Table 7), which is quite different from the number of pathways we found previously in other species in the same genus; for example, *C. pseudotuberculosis *strains 1002 and C231 in which 105 and 104 Biosynthesis pathways were predicted, respectively [25]. The number of Degradation/Utilization/Assimilation pathways predicted in the DSM 7109 and DSM 7111 genomes where similar, 70 pathways in DSM 7109 and 66 pathways in DSM 7111 (Table 7).

**Table 7:** Numbers of pathways in *C. urealyticum *strains DSM 7109 and DSM 7111.

Pathway class	DSM 7109	DSM 7111
**Biosynthesis**	139	174
-Amines and Polyamines Biosynthesis	4	5
-Amino Acids Biosynthesis	27	32
-Aminoacyl-tRNA Charging	3	3
-Aromatic Compounds Biosynthesis	2	4
-Carbohydrates Biosynthesis	6	9
-Cell Structures Biosynthesis	4	5
-Cofactors, Prosthetic Groups, Electron Carriers Biosynthesis	34	40
-Fatty Acids and Lipids Biosynthesis	6	8
-Metabolic Regulators Biosynthesis	1	1
-Nucleosides and Nucleotides Biosynthesis	18	25
-Other Biosynthesis	5	5
-Secondary Metabolites Biosynthesis	4	3
**Degradation/Utilization/Assimilation**	70	66
-Alcohols Degradation	1	4
-Amines and Polyamines Degradation	5	3
-Amino Acids Degradation	18	17
-Aromatic Compounds Degradation	3	2
-C1 Compounds Utilization and Assimilation	1	3
-Carbohydrates Degradation	4	2
-Carboxylates Degradation	5	6
-Degradation/Utilization/Assimilation - Other	2	1
-Fatty Acid and Lipids Degradation	3	4
-Inorganic Nutrients Metabolism	10	8
-Nucleosides and Nucleotides Degradation	6	5
-Protein Degradation	2	2
-Secondary Metabolites Degradation	2	2
-Steroids Degradation	2	2
**Generation of Precursor Metabolites and Energy**	16	25

**Total**	185	216

On further analysis, we found that the DSM 7109 and DSM 7111 genomes had 25 and 57 unique metabolic pathways (Table S1, additional file 1), respectively, even though both the strains were isolated from humans and caused the same symptoms [6,7].

## Conclusions

To our knowledge, this is the first comparative genomic study using the complete genome sequences of two *C. urealyticum *strains, DSM 7109 and DSM 7111. Our analyses provided insights into the genome architecture and the gene content of this species. We found that the *C. urealyticum *DSM 7111 genome was 50 kb shorter than the *C. urealyticum *DSM 7109 genome. This difference in genome size may be linked to the large number of genomic islands (26 for each genome) predicted for both genomes. The genomic islands may have resulted from the horizontal transfer of genes, leading to the acquisition of many strain-specific genes. We detected a high number of strain-specific genes in the two genomes compared with the low number of species-specific genes that have been reported in previous studies of others species of Corynebacterium [25,30]. The horizontal transfer of genes may also explain why *C. urealyticum *is multi-drug resistant; i.e., it has received virulence genes by horizontal transfer [4,5].

*C. urealyticum *is a pathogenic opportunistic bacteria although it showed the *spaDEF *operon (virulence factor), with a structure similar to that of pathogenic species like *C. diphtheriae *and *C. ulcerans*. This operon encodes an adhesive pilus responsible for facilitating the adhesion of the pathogen to host cells [28,43].

This comparative genomic study of two *C. urealyticum *strains provides a basis using reverse vaccinology to predict new antigenic targets. However, additional *C. urealyticum *strains will have to be studied to create effective vaccines against this bacterium.

## Competing interests

The authors declare that they have no competing interests.

## Authors' contributions

LCG, SCS, and VA conceived the study and designed the experiments. LCG, SCS, ET, and RTJR performed the experiments. LCG, SCS, and TRJR analyzed the data. SCS, JB, AS, DB, and VA contributed the materials and analyses tools. AS, DB, JB, and VA provided insights and technical inputs. LCG wrote the paper and SCS, ET, and DB edited the manuscript. All authors read and approved the final manuscript.

## Supplementary Material

Additional file 1 Table S1 *C. urealyticum *strain-specific pathways. Click here for file

## Declarations

The publication of this work is funded by CNPq (National Counsel of Technological and Scientific Development), FAPEMIG (Foundation for Research Support of the State of Minas Gerais) and CAPES (Coordination for the Improvement of Higher Education Personnel) number SC-1000675.

This article has been published as part of *BMC Genomics *Volume 16 Supplement 5, 2015: Proceedings of the 10th International Conference of the Brazilian Association for Bioinformatics and Computational Biology (X-Meeting 2014). The full contents of the supplement are available online at http://www.biomedcentral.com/bmcgenomics/supplements/16/S5.
