# Supplementary material for: Genome informatics and vaccine targets in Corynebacterium urealyticum using two whole genomes, comparative genomics, and reverse vaccinology
Source: BMC Genomics. 2015 May 26;16(Suppl 5):S7. doi: 10.1186/1471-2164-16-S5-S7 (PMC4460590; doi:10.1186/1471-2164-16-S5-S7)
Supplement: Additional file 1 — Table S1 C. urealyticum strain-specific pathways. [file 1471-2164-16-S5-S7-S1.pdf]

Table S1, additional file 1- *C. urealyticum* strain-specific pathways

**Pathway Class -** Pathway name	**DSM 7109**	**DSM 7111**
**Biosynthesis - Amines and Polyamines Biosynthesis**		
-glycine betaine biosynthesis II (Gram-positive bacteria)	absent	present
**Biosynthesis - Amino Acids Biosynthesis**		
-alanine biosynthesis I	absent	present
-alanine biosynthesis II	absent	present
-arginine biosynthesis II (acetyl cycle)	present	absent
-cysteine biosynthesis I	absent	present
-glutamine biosynthesis I	absent	present
-glycine biosynthesis III	absent	present
-phenylalanine biosynthesis I	absent	present
**Biosynthesis - Aromatic Compounds Biosynthesis**		
-2,3-dihydroxybenzoate biosynthesis	absent	present
-4-hydroxyphenylpyruvate biosynthesis	absent	present
**Biosynthesis - Carbohydrates Biosynthesis**		
-UDP-α-D-glucuronate biosynthesis (from UDP-glucose)	absent	present
-UDP-D-galactose biosynthesis	absent	present
-UDP-glucose biosynthesis	absent	present
**Biosynthesis - Cell Structures Biosynthesis**		
-UDP-α-D-glucuronate biosynthesis (from UDP-glucose)	absent	present
**Biosynthesis - Cofactors, Prosthetic Groups, Electron Carriers Biosynthesis**		
-4-methyl-5(β-hydroxyethyl)thiazole salvage (yeast)	absent	present
-biotin biosynthesis from 7-keto-8-aminopelargonate	present	absent
-chlorophyllide a biosynthesis I (aerobic, light-dependent)	absent	present
-chlorophyllide a biosynthesis II (anaerobic)	absent	present
-chlorophyllide a biosynthesis III (aerobic, light independent)	absent	present
-cob(II)yrinate a,c-diamide biosynthesis I (early cobalt insertion)	absent	present
-di-trans,poly-cis-undecaprenyl phosphate biosynthesis	absent	present
-mycothiol biosynthesis	absent	present
-mycothiol oxidation	present	absent
-NAD phosphorylation and dephosphorylation	present	absent
-pyridoxal 5'-phosphate biosynthesis II	present	absent
-pyridoxal 5'-phosphate salvage I	absent	present
-siroheme biosynthesis	absent	present
-tetrapyrrole biosynthesis I (from glutamate)	absent	present
-thiamin diphosphate biosynthesis III (Staphylococcus)	present	absent
-thiazole biosynthesis II (Bacillus)	present	absent
-thioredoxin pathway	absent	present
-[2Fe-2S] iron-sulfur cluster biosynthesis	absent	present
**Biosynthesis - Fatty Acids and Lipids Biosynthesis**		
-(KDO)2-lipid A biosynthesis I	absent	present
-cardiolipin biosynthesis III	absent	present
**Biosynthesis - Nucleosides and Nucleotides Biosynthesis**		

-adenosine deoxyribonucleotides de novo biosynthesis I	absent	present
-adenosine deoxyribonucleotides de novo biosynthesis II	absent	present
-adenosine ribonucleotides de novo biosynthesis	absent	present
-CMP phosphorylation	absent	present
-guanosine deoxyribonucleotides de novo biosynthesis I	absent	present
-guanosine deoxyribonucleotides de novo biosynthesis II	absent	present
-guanosine ribonucleotides de novo biosynthesis	absent	present
-purine and pyrimidine metabolism	present	absent
-purine deoxyribonucleosides salvage	absent	present
-pyrimidine deoxyribonucleotide phosphorylation	absent	present
-pyrimidine deoxyribonucleotides de novo biosynthesis I	absent	present
-pyrimidine deoxyribonucleotides de novo biosynthesis II	present	absent
-pyrimidine deoxyribonucleotides de novo biosynthesis II	absent	present
-pyrimidine ribonucleotides salvage	present	absent
-queuosine biosynthesis	present	absent
**Biosynthesis - Secondary Metabolites Biosynthesis**		
-fluoroacetate and fluorothreonine biosynthesis	present	absent
**Degradation/Utilization/Assimilation - Alcohols Degradation**		
-ethanol degradation I	absent	present
-ethanol degradation II	absent	present
-glycerophosphodiester degradation	absent	present
**Degradation/Utilization/Assimilation - Amines and Polyamines Degradation**		
-4-aminobutyrate degradation I	present	absent
-4-aminobutyrate degradation II	present	absent
**Degradation/Utilization/Assimilation - Amino Acids Degradation**		
-alanine degradation III	absent	present
-proline degradation	present	absent
-threonine degradation III (to methylglyoxal)	present	absent
**Degradation/Utilization/Assimilation - Aromatic Compounds Degradation**		
-protocatechuate degradation III (para-cleavage pathway)	present	absent
**Degradation/Utilization/Assimilation - C1 Compounds Utilization and Assimilation**		
-formate oxidation to CO2	absent	present
-formate reduction to 5,10-methylenetetrahydrofolate	absent	present
**Degradation/Utilization/Assimilation - Carbohydrates Degradation**		
-acetoin degradation	present	absent
-galactose degradation I (Leloir pathway)	present	absent
**Degradation/Utilization/Assimilation - Carboxylates Degradation**		
-glutaryl-CoA degradation	absent	present
**Degradation/Utilization/Assimilation - Other**		
-acrylonitrile degradation I	absent	present
-nicotine degradation I	present	absent
-nicotine degradation II	present	absent
**Degradation/Utilization/Assimilation - Fatty Acid and Lipids Degradation**		
-fatty acid β-oxidation VI (peroxisome)	absent	present
**Degradation/Utilization/Assimilation - Inorganic Nutrients Metabolism**		
-phosphate acquisition	present	absent

-phosphate utilization in cell wall regeneration	present	absent
**Degradation/Utilization/Assimilation - Nucleosides and Nucleotides Degradation**		
-guanosine nucleotides degradation III	present	absent
**Generation of Precursor Metabolites and Energy**		
-aerobic respiration (cytochrome c) (yeast)	absent	present
-formate oxidation to CO2	absent	present
-glycerol-3-phosphate shuttle	absent	present
-glycolysis I (from glucose-6P)	absent	present
-glycolysis IV (plant cytosol)	absent	present
-glycolysis VI (metazoan)	absent	present
-photosynthesis light reactions	present	absent
-pyruvate fermentation to acetate II	absent	present
-pyruvate oxidation pathway	absent	present
-succinate to cytochrome bd oxidase electron transfer	absent	present
-TCA cycle VII (acetate-producers)	absent	present
